# Social media use, gaming and quality of life among adolescents in Norway: results from the Young-HUNT study

**DOI:** 10.1186/s12955-025-02428-1

**Published:** 2025-10-31

**Authors:** Annette Løvheim Kleppang, Curt Hagquist, Tonje Holte Stea, Marja Leonhardt, Tore Bonsaksen, Lars Lien, Anne Mari Steigen

**Affiliations:** 1 Department of Public Health and Sport Sciences, University of Inland Norway, P.O. Box 400, Elverum, 2418 Norway; 2 Department of Education and Special Education, University of Gothenburg, Gothenburg, Sweden; 3 Department of Health and Nursing Science, University of Agder, Kristiansand, Norway; 4 Research Centre for Substance Use Disorders and Metal Illness, Inland Hospital Trust, Brumunddal, Norway; 5 Section for Clinical Addiction Research, Oslo University Hospital, Oslo, Norway; 6 Department of Health and Nursing Sciences, University of Inland Norway, Elverum, Norway; 7 Department of Health, Faculty of Health Sciences, VID Specialized University, Stavanger, Norway

**Keywords:** Adolescents, Social media use, Gaming and quality of life

## Abstract

**Background:**

The use of screens, especially mobile devices like tablets and smartphones has increased over the last years and have become an integral to daily life. Adolescents today spend a lot of time using screens both at school and outside school. Identifying possible associations between gaming, social media use and quality of life (QoL) may inform the development of public health guidelines targeting adolescent behaviour. The purpose of this study is to examine the association between gaming, social media use and quality of life (QoL) among adolescents, and whether sex, spending time with friends or psychological distress moderate these associations.

**Methods:**

This study was based on cross-sectional data from the Trøndelag Health study (Young-Hunt4), collected in 2017–2019. The target group comprised 8066 adolescents (13–19 years). QoL was measured with the Norwegian version of the Inventory of Life Quality (ILC). Electronic media use was measured using the two variables social media use and gaming. Multiple linear regression was used to analyse the association between social media use, gaming, and QoL.

**Results:**

Results showed a negative association between gaming (> 3 h per day) after school and quality of life (b = -0.26) and social media use (> 3 h per day) after school and quality of life (b = -0.20). Similar negative associations were observed between more time spent on gaming (> 3 h per day) during weekends and quality of life (b = -0.33) and more time spent on social media (> 3 h per day) during weekends and quality of life (b = -0.16). An interaction effect was observed between sex and social media use (after school), between sex and gaming (after school and during weekends), and between psychological distress and gaming during weekends.

**Conclusions:**

More than three hours spent on gaming or social media per day, both during weekdays and weekends, was negatively associated with QoL. Sex moderated these associations; however, for social media use this was shown only after school. Additional research is needed to further explore these associations.

## Background

The World Health Organization (WHO) defines Quality of Life (QoL) as ‘the individual’s perception of their position in life in the context of the culture and value systems in which they live, and in relation to their goals, expectations, standards and concerns’ [1]. There is a large number of definitions of QoL [2], and the concept is multidimensional and complex [3, 4]. In the present study, we define QoL as adolescent’s perceived well-being and satisfaction with life that is best evaluated by the adolescent, with regard to several life domains such as physical, psychological, and social functioning as well in the family, school, and leisure time [2, 5]. To enhance and better understand QoL, it is important to examine factors associated with QoL in adolescence.

Despite the rapid evolution of media, the fundamental aspects of adolescent development remain unchanged. Adolescence is a time of intense self-discovery and identity formation, during which individuals strive to establish a sense of self that is independent of their parents. Contemporary adolescents allocate a greater portion of their time to gaming and social media in comparison to earlier generations [6, 7]. With the growing integration of technology into our daily lives, adolescents are encountering electronic products at increasingly younger ages, leading to a rise in their screen time, including time spent with gaming and on social media. Higher screen time among youth, coinciding with a decrease in their engagement with nature, impact their mental health and overall well-being [8]. Whereas girls are predominantly active on social media`, boys tend to be more engaged in digital- or video gaming activities [9, 10]. Moreover, adolescent girls generally utilize social media to communicate with their peers and to strengthen pre-established relationships, while boys on the other hand more actively seek out to encounter new acquaintances and make new friends online [11].

Adolescence is an important period in life during which the opportunities for good health and future patterns of adult’s health are established [12]. The deleterious impacts of social media use and gaming on adolescents’ mental health have been documented. These include symptoms of depression and anxiety [13, 14], feelings of loneliness [15, 16] and a diminished quality of life [17]. Social media use has also been associated with lower self-esteem and life-satisfaction [18], and internet gaming has been found to be associated with lower psychological QoL [19]. A systematic review corroborated an adverse impact of smartphone use on mental well-being [20]. Both ends of the digital technology usage spectrum - low and excessive - have been linked to decreased well-being, while moderate use appears to foster increased well-being [21]. Notably, a systematic review of 34 longitudinal studies found gender differences in the association between screen time and internalized mental health issues [22]. However, the gender differences shown by S Tang, A Werner-Seidler, M Torok, AJ Mackinnon and H Christensen [22] were largely inconsistent, as some studies reported a relationship between screen time and internalising mental health outcomes among males, but not females, whereas other studies reported the opposite.

Excessive use of social media and gaming may lead to a decrease in face-to-face social interactions and may impair the development of social skills in adolescents. However, the impact of screen time on QoL appears to depend on the type of activity. Passive screen activities such as simply observing others’ content and procrastinating have been related to more negative effects on mental wellbeing, whereas engaging actively in social media by chatting, posting, liking, or sharing content has been linked to more positive outcomes [21]. Given the risk of mental health problems that emerge in adolescents potentially continuing into adulthood, identifying lifestyle behaviours that are associated with better QoL may provide an opportunity to ensure that young people have a healthier progression through adolescence.

Furthermore, mental health problems have shown to have negative effects on health related QoL, while social support is a protective factor [23]. Additionally, the parents’ sociodemographic status is important for children’s QoL [24], while limited access to material and social resources can diminish adolescents’ health related quality of life (HRQoL) [25]. Moreover, having multiple reciprocal friendships has been demonstrated to enhance QoL [26]. Gender differences in QoL among adolescents have also been reported, indicating that girls report a lower level of QoL than boys [27, 28], and there is also evidence that suggest quality of life is lower during adolescence compared to earlier childhood years [29].

The relationship remains unclear when it comes to the impact of gaming and social media use on QoL, it remains unclear whether specific forms of screen time are differentially associated with QoL. While screens have undeniably made information more accessible and facilitated connections across distances, there is growing concern about their impact on adolescents’ health and overall quality of life. Despite the research on screen use and mental health problems, there remains a significant gap in understanding the association between gaming, social media use and QoL. Acknowledging the multifaceted nature of QoL, which spans physical, psychological, and social dimensions, the present study adds to the current literature by differentiating between gaming and social media use and examining their independent associations to QoL in a representative sample of adolescents. Additionally, this study explores whether factors like sex, spending time with friends or psychological distress modify these associations.

## Methods

### Data collection and study population

The present study was a part of the Trøndelag Health Study (HUNT) [30, 31]. All adolescents aged 13–19 years living in the county of Trøndelag in Norway were invited to participate in the Young-HUNT4 survey (2017–2019), of whom 8066 adolescents participated (76.0% participation rate). The Young-HUNT4 survey utilised a self-report questionnaire, covering a range of health-related topics. The adolescents filled in the questionnaire on electronic tablets. Most of them were recruited through schools and participated while at school. Participation was voluntary, and the respondents were informed that they could withdraw from the study at any time. The HUNT study was a collaboration between the HUNT Research Centre (Faculty of Medicine and Health Sciences, Norwegian University of Science and Technology NTNU), Trøndelag County Council, Central Norway Regional Health Authority and the Norwegian Institute of Public Health.

## Measures section 2

### Quality of life (QoL) section 3

Quality of life (QoL) was measured with the Norwegian version [32] of the Inventory of Life Quality in children and adolescents (ILC). The ILC is a brief measure to assess QoL in children and adolescents aged 6–18 years [33]. The scale consists of seven items; one item assesses the overall QoL, and six items address the adolescents’ physical and mental health, autonomy in play, and perceived relationship with family and friends, and functioning at school. The seven questions have five response categories: ‘very good (1)‘, ‘quite good (2)‘, ‘neither good nor bad (3)‘, ‘quite bad (4)‘ and ‘very bad (5)‘. A QoL score (ranges from 0–28) is calculated across all items, where a higher scores indicate better QoL (LQ_0−28_) [32, 33]. The Norwegian version of the ILC has been examined and shown to function satisfactory [34].

In the present study, the *Rasch Measurement Theory* [35, 36] was used to examine the psychometric properties of the ILC scale. The scale shows good reliability (Person Separation Index: 0.79), indicating good separation of persons. The persons were relatively well targeted as a whole, but the person’s locations were skewed, with a positive mean (1.88), implying that item thresholds capturing persons located further to the right (higher levels of QoL) of the scale are missing. Two of the items, item 1, ‘school performance’ and item 2 “family relationship “, indicate misfit, and some items work differently for boys and girls. Additionally, three items, item 2, 3 and 7 showed reversed threshold ordering, which indicates problems with categorization of the items. To address these problems, we collapsed category 2 and 3 and examined the scale with four response categories. All the items had ordered response categories after collapsing the two categories. The ILC scale works better with four response categories and was therefore chosen to use in the present study. In the Rasch analysis, the non-linear raw scores are transformed to person estimates on a linear interval logit scale on which each person is allocated a location (logit) value. These person estimates were used in the statistical analysis in the present study. Higher values on the scale indicate a higher degree of QoL.

We conclude that the ILC has the potential to measure QoL among adolescents but clearly, there is room for improvements.

## Gaming and social media use section 3

Electronic media use was assessed via two variables: social media use and gaming. Social media use was measured by asking: ‘In your spare time, how many hours per day do you spend on social media or surf/chat on the internet on weekdays (Monday to Friday) and during weekends (Saturday and Sunday)‘.Gaming was measured by asking: ‘In your spare time, how many hours per day do you play video games (on PC, game console, tablet, cell phone, etc.) on weekdays and during weekends‘, and for both questions, the respondents were asked to indicate one response each for weekdays (spare time) and weekends. The two questions have six response categories for both weekdays and the weekends: ‘Not at all (1)‘, ‘Less than half an hour a day (2)‘, ‘1/2–1 h a day (3)‘, ‘2–3 h a day (4)‘, ‘4–6 h a day (5)‘ and approximately ‘7 hours or more a day (6)‘. Social media use and gaming were dichotomised into > 3 hours per day and ≤ 3 hours per day.

## Psychological distress section 3

The Hopkins Symptoms Checklist-10 (HSCL-10) is used to measure psychological distress. The scale consists of 10 questions about symptoms of depression and anxiety. The 10 questions have four response categories: ‘Not at all’, ‘A little’, ‘Quite a bit’ and ‘Extremely’. The responses are summarized across all items, and the mean score is used to measure psychological distress. An average score ≥ 1.85 is considered a valid cut-off value for prediction of mental distress [37]. The scale has been examined among Norwegian adolescents, showing good reliability. On the whole, the items work well [38].

### Spent time with friends after school hours section 3

Spending time with friends was measured by asking ‘How often have you done any of these activities? ‘Hang out with friends after school hours?’. The response options included: ‘Never (1)’, ‘1–3 times a month or less (2)’, ‘About once a week (3)’, ‘2–3 times a week (4)’ and ‘4 times a week or more (5)’. Hang out with friends after school hours was dichotomised into about once a week and 2–3 times a week and more.

## Age and sex of the adolescents section 3

The age of the respondents in YoungHUNT4 varied between 12.7 and 21.8 years and was dichotomised as ≤ 16.1 years, and > 16.1 years (reference category). Sex was categorized as females and males.

## Socio-economic status (SES) section 3

Family economy was measured using the question whether they felt their family was better or worse off economically than others. Response options were worse off (1), the same (2), better off (3). Education plans were categorized as higher education and no higher education.

### Statistical analysis

Independent *t-tests* were conducted to study differences in QoL across sex and other dichotomised variables. When studying differences in QoL across groups, *a one-way analysis of variance* (*ANOVA*) was used. Multiple linear regression was conducted to explore the association between screen use and QoL, controlled for sex, age, family economy, psychological distress and spending time with friends. The independent variables were constructed and analysed using dummy-regressor coding. Interaction analyses were used to examine whether sex and spending time with friends affected the strength of the relationship between electronic media use and QoL. Possible interaction effects were examined using an incremental F-test [39], contrasting models with and without interaction terms. The main effect model included screen use, sex, age, family economy, psychological distress and spending time with friends as independent variables and was tested against models also including interactions between social media use after school by sex, social media use after school by spending time with friends and social media use after school by psychological distress. Similar interaction analyses were carried out for social media use during weekends, gaming after school and gaming during weekends. Only the significant interactions are presented in the results section.

Statistical significance was assumed at *p* < 0.05. *The linear regression* analysis were conducted using the Statistical Package for Social Sciences (SPSS), version 28 (IBM Corporation), and the software RUMM2030 + was used to perform the *Rasch analysis* [40].

## Results

A total of 8066 adolescents responded to the survey. The proportion of males and females and the age cohort was approximately equal. Among the adolescents, 44.9% reported planning to pursue higher education, and most of the adolescents reported a family economy like most others (70.4%). The distribution of spending time with friends was similar to not spending time with friends, and 25.6% of the adolescents reported higher level of psychological distress.

Table 1 shows the characteristics of the sample and QoL, across levels of person factors, spending time with friends, psychological distress.

**Table 1:** Sample characteristics and Qol (in logits) across levels of personal and emotional factors, social media use and gamming

Characteristics	n (%)	QoL mean (SD)	P value	Cohen's (d)
Number of participants	8066	1.98 (1.69)		
Sex				
Females	4108 (50.9)	1.66 (1.65)	< 0.001	-0.398
Males	3958 (49.1)	2.32 (1.66)		
Age				
≤ 16.1	3958 (49.1)	2.14 (1.69)	< 0.001	0.185
>16.1	4108 (50.9)	1.82 (1.67)		
Family economy				
Better economy	1636 (20.3)	2.38 (1.63)	< 0.001	0.048*
Like most others	5682 (70.4)	1.99 (1.65)		
Worse economy	629 (7.8)	0.83 (1.65)		
Education plans				
Higher education	3625 (44.9)	2.01 (1.61)	0.059	-0.043
No higher education	4329 (53.7)	1.94 (1.75)		
Time spent with friends				
About once a week	3750 (46.5)	1.67 (1.68)	< 0.001	-0.403
More than 2 times a week	4020 (49.8)	2.26 (1.65)		
Psychological distess				
Higher level of psychological distress	2063 (25.6)	0.60 (1.32)	< 0.001	1.270
Lower level of psychological distress	5762 (71.4)	2.47 (1.52)		
Social media use				
> 3 hours per day, after school	2552 (31.6)	1.55 (1.72)	< 0.001	0.383
≤ 3 hours per day, after school	5193 (64.4)	2.19 (1.63)		
> 3 hours per day, during weekends	3364 (41.7)	1.62 (1.69)	< 0.001	0.381
≤ 3 hours per day, during weekends	4244 (52.6)	2.25 (1.63)		
Gaming				
> 3 hours per day, after school	1529 (19.0)	1.54 (1.72)	< 0.001	0.329
≤ 3 hours per day, after school	6249 (77.5)	2.09 (1.66)		
> 3 hours per day, during weekends	2704 (33.5)	1.73 (1.68)	< 0.001	0.226
≤ 3 hours per day, during weekends	4971 (61.6)	2.11 (1.68)		

QoL: quality of life (Person location estimates in logits). Higher values indicate higher QoL. Missing values represented 0 % for age and sex, 4.5% for QoL, 1.5 % for family economy, 1.4 % for education plans, 3.7 % for spending time with friends, 3% for psychological distress, 3.6% for gaming after school, 4.8% during weekend, and 4% for social media use after school, 5.7% during weekends. Differences across dichotomized person factors, emotional factors and social media use and gaming were analysed using the independent sample t-test. Differences in QoL across family economy was analysed using one-way analysis of variance (ANOVA). *Eta squared

Quality of life (QoL) was examined across various person factors, psychological distress levels, and patterns of gaming and social media use. Higher QoL was statistically significantly associated with better family economy, lower psychological distress, being 16 years old or younger, being male, and limiting gaming or social media use to three hours or less per day. The magnitude of the mean differences for these factors were small (ranging from 0.2 to 0.3), except for psychological distress, which showed a large effect size (≥ 0.8).

Table 2 shows the results of the analysis of the association between social media use, gaming, sex, age, family economy, time spent with friends and psychological distress.

**Table 2:** Multiple linear regression analysis of QoL in relation to social media use and gaming, adjusting for sex, age, family economy, psychological distress and time spent with friends (Young-Hunt4 study, adolescents aged 13–19 years)

	Model A			Model B			Model C			Model D			Model E			Model F		
	b (SE)	β	CI	b (SE)	β	CI	b (SE)	β	CI	b (SE)	β	CI	b (SE)	β	CI	b (SE)	β	CI
Electronic media use																		
Social media use																		
More than 3 hours, after school	−0.20 (0.05)	−0.06***	−0.30, −0.10				−0.13 (0.05)	−0.04*	−0.23, −0.03	−0.22 (0.06)	−0.06***	−0.34, −0.10	−0.14 (0.05)	−0.04**	−0.24, −0.04	−0.13 (0.05)	−0.04*	−0.24, −0.03
More than 3 hours, during weekends	−0.16 (0.05)	−0.05**	−0.26, −0.07				−0.13 (0.05)	−0.04**	−0.23, −0.03	−0.14 (0.05)	−0.04**	−0.24, −0.04	−0.13 (0.05)	−0.04**	−0.22, −0.03	−0.13 (0.05)	−0.04*	−0.23, −0.04
Gaming																		
More than 3 hours after school				−0.26 (0.05)	−0.06***	−0.37, −0.16	−0.21 (0.06)	−0.05***	−0.32, −0.10	−0.21 (0.06)	−0.05***	−0.31, −0.10	−0.10 (0.08)	−0.02	−0.25, −0.05	−0.23 (0.06)	−0.05***	−0.33, −0.12
More than 3 hours during weekends				−0.33 (0.05)	−0.09***	−0.42, −0.24	−0.31 (0.05)	−0.09***	−0.41, −0.22	−0.31 (0.05)	−0.09***	−0.40, −0.22	−0.33 (0.05)	−0.09***	−0.42, −0.24	−0.20 (0.07)	−0.06**	−0.32, −0.07
Sex																		
Males	0.14 (0.04)	0.04***	0.07, 0.21	0.30 (0.04)	0.09***	0.23, 0.38	0.26 (0.04)	0.08***	0.19, 0.34	0.20 (0.04)	0.06***	0.11, 0.28	0.30 (0.04)	0.09***	0.22, 0.38	0.33 (0.05)	0.10***	0.24, 0.41
Age																		
>16.1	−0.10 (0.03)	−0.03**	−0.17, −0.04	−0.18 (0.03)	−0.05***	−0.24, −0.11	−0.15 (0.03)	−0.05***	−0.22, −0.08	−0.15 (0.03)	−0.05***	−0.22, −0.09	−0.15 (0.03)	−0.04***	−0.22, −0.08	−0.15 (0.03)	−0.04***	−0.22, −0.08
Family economy																		
Worse economy	−0.10 (0.03)	−0.04**	−0.15, −0.05	−0.10 (0.03)	−0.04***	−0.15, −0.05	−0.10 (0.03)	−0.04**	−0.15, −0.04	−0.09 (0.03)	−0.04**	−0.15, −0.04	−0.10 (0.03)	−0.04***	−0.15, −0.04	−0.10 (0.03)	−0.04***	−0.15, −0.04
Time spent with friends																		
More than 2 times a week	0.45 (0.03)	0.13***	0.39, 0.52	0.39 (0.03)	0.12***	0.33, 0.46	0.42 (0.03)	0.13***	0.36, 0.49	0.42 (0.03)	0.13***	0.36, 0.49	0.42 (0.03)	0.12***	0.35, 0.49	0.42 (0.03)	0.12***	0.35, 0.49
Psychological distess																		
Higher level of psychological distress	−1.68 (0.04)	−0.44***	−1.76, −1.60	−1.69 (0.04)	−0.44***	−1.77, −1.61	−1.65 (0.04)	−0.43***	−1.73, −1.57	−1.64 (0.04)	−0.43***	−1.72, −1.56	−1.65 (0.04)	−0.43***	−1.73, −1.57	−1.65 (0.04)	−0.43***	−1.73, −1.57
Interaction sex by electronic media use																		
Male*Social media use after school										0.22 (0.07)	0.04**	0.08, 0.36						
Male*Gaming after school													−0.17 (0.09)	−0.03*	−0.35, 0.00			
Male*Gaming during weekends																−0.19 (0.08)	−0.05*	0.34, −0.04

Dependent variable is QoL level with four response categories (Person location estimates in logits), higher level indicate better QoL; *b* unstandardized beta weights, *β* standardized beta weights, *CI* standardized confidence intervals, * p < 0.05, ** p < 0.01, ***p < 0.001

Model A = Social media use analysed seperately, adjusting for sex, age, family economy, psychological distress and spending time with friends. Model fit: adjusted R^2^ =.27; F= 392.56

Model B = Gaming, adjusting for sex, age, family economy, psychological distress and spending time with friends. Model fit: adjusted R2 =.28; F= 413.36

Model C = Social media use and gaming, adjusting for sex, age, family economy, psychological distress and spending time with friends. Model fit: adjusted R2 =.29; F= 321.71

Model D = Social media use and gaming, adjusting for sex, age, family economy, psychological distress and spending time with friends, aldo including the interaction term male*social media use after school. Model fit: adjusted R2 =.29; F= 290.75

Model E = Social media use and gaming, adjusting for sex, age, family economy, psychological distress and spending time with friends, aldo including the interaction term male*gaming after school. Model fit: adjusted R2 =.29; F= 290.06.

Model F = Social media use and gaming, adjusting for sex, age, family economy, psychological distress and spending time with friends, aldo including the interaction term male*gaming during weekends. Model fit: adjusted R2 =.29; F= 290.40

*Results in Table 2 show an inverse association between gaming (> 3 h per day) and QoL (b = -*0.26) and social media use (> 3 h per day) and QoL (b = −0.20) after school. Similar inverse associations are observed between gaming (> 3 h per day) and QoL (b = −0.33) and social media use (> 3 h per day) and QoL (b = −0.16) during weekends. These associations remained stable after controlling for all four variables (social media use and gaming after school and during weekends).

An interaction effect was observed between sex and gaming, meaning that the association between gaming and QoL differs for males and females. Separate analysis for male and female showed significant associations for both sexes in the weekend (male: b = −0.39; female: b = −0.23), and after school (male: b = −0.27; female: b = −0.28). The same effect was observed for sex and social media use after school, meaning that the association between social media use after school and QoL differs for males and females. After separate analysis for male and females, a significant association was only observed for females, not for males (male: b = −0.01; female: b = −0.33). Moreover, an interaction effect was observed between psychological distress and gaming in the weekends, meaning that the association with QoL was not significant for those with psychological distress while it was significant for those with no psychological distress. These patterns were confirmed by incremental F-test. (see Table 3).

There was no interaction effect between electronic media use and spending time with friends (not shown in Table 2).

## Discussion

Our results showed that adolescents’ frequent use of social media and gaming was significantly associated with lower QoL, however, the associations were weak. These results are consistent with findings of previous research linking more time on screens to lower HRQoL [41, 42].

More frequent social media use was significantly associated with lower QoL in the present study after adjustment for confounders. Previous studies have also indicated that prolonged social media usage and engagement with numerous social media platforms are associated with lower HRQoL [43]. Furthermore, the impact of time spent on social media on psychological well-being have been reported to be negative, but very small [44], while moderate digital technology use appears to foster increased well-being [21]. It is possible that a high overall social media consumption also indicates a high level of social interaction with friends [45, 46], which may account for a positive contribution to QoL. In contrast a randomized control trial among students did not find significant causal effects of social media usage on well-being [47]. In line with this, recent large-scale preregistered studies have reported an absence of substantial or practically meaningful correlations between adolescents’ digital technology usage and well-being [48]. Thus, the complex nature of social media use makes it difficult to fully understand how it is various aspects may contribute to the young person’s QoL. However, based on this study, we note that the overall association between time spent on social media and QoL was negative, in line with several studies.

Similarly, we found that more frequent gaming was associated with lower QoL. Spending time on gaming has also been negatively associated with psychological wellbeing in adolescence [49]. A cross-sectional study employing objective measures of video gaming, discovered a positive relationship between video gaming and well-being [50]. Playing video games provides many emotional, cognitive and social benefits [51, 52]. Playtime may influence well-being depending on players’ motivations and how the game satisfies basic needs. Games are inherently social; they may shape relationships, influence feelings of social support, and contribute to overall well-being [53]. However, they may also take time away from real-life social relationships and schoolwork and can be the root of parental concern and family conflicts, all of which in turn can influence lower QoL. Both sleep duration and physical activity frequency have been reported to mediate the relationship between gaming and psychological wellbeing [49]. The possibility of a reversed causal chain should also be mentioned: adolescents with lower QoL may be more inclined to seek stress relief and diversion through an alternative reality in gaming, compared to their counterparts with higher QoL.

In the present study, an interaction effect was observed between sex and social media use after school, meaning that the association between social media use and QoL differed for males and females. Separate analysis for males and females showed significant associations only for females, not for males. Our results are consistent with previous studies that found social media use to be more strongly associated with measures of well-being for girls compared to boys [54, 55]. A possible explanation could be the sex differences in use of social media that have been reported in the literature. Previous research has shown that girls report greater use of social media for social compensation, emotion bonding and appearance validation, than boys, who report greater competitive activity bonding [56]. Furthermore, results from a study among adolescents showed sex differences in screen use, with females predominantly using the social media sites TikTok, Snapchat, Instagram, and Pinterest while males predominantly used YouTube [46]. Moreover, in Norwegian adolescents, an increased use of Facebook, Instagram and TikTok have been linked to increased focus on self-presentation, which was associated with reduced QoL, and the associations were stronger for girls than for boys [57].

An interaction effect was observed between psychological distress and gaming in the weekend, indicating that the association with QoL was significant only for individuals without psychological distress. For those experiencing psychological distress, the relationship was not significant. This may be explained by the possibility that individuals with psychological distress could diminish or counteract the potential benefits of gaming on QoL. In contrast, for those without psychological distress, gaming may have a positive impact on their well-being. Interestingly, a previous study on adolescents found that psychological distress mediated the relationship between internet gaming disorders and QoL [58].

### Strength and limitations

A major Strength of this study is the large sample size in combination with a high response rate. The data used in this study were collected in 2017–2019 and provide an up-to-date description of key aspects of young people’s lives. However, development in the digital world occur rapidly and there might have been changes in both use and attitudes towards social media and gaming among adolescents in the last 6–8 years influencing the association with QoL. This study also has additional limitations. This is a cross-sectional study, which precludes inferences about causal relationships. QoL may not only work as an outcome but may also act as an exposure that is hampering social media use and gaming. Furthermore, the use of self-reported measures may have introduced unidentified misclassifications or measurement errors. A general trend is to overestimate time used on screens [59], like smartphone use [60] and gaming [50, 61]. To our knowledge, no evaluation has been performed in the HUNT survey regarding self-reporting of social media use and gaming. Furthermore, the associations found between social media use, gaming and QoL might be influenced by other factors that were not controlled for in the present study. For example, our data did not include information regarding the content of gaming and social media, and a previous study of Norwegian adolescents found negative social media events to be more strongly related to depressive symptoms than the time spent on social media [62].We had no information about what types of games adolescents were playing or what they were doing on social media. Thus, our results should be interpreted with caution.

## Conclusions

Results from the present study indicate that more frequent use of social media and gaming were associated with a lower level of QoL in adolescents, and that these associations varied by sex. These findings have implications for how digital media consumption is understood in relation to adolescent well-being. Our results may inform public health strategies and educational policies aimed at promoting healthier digital habits and be useful for general practice in promoting adolescents’ QoL. The positive effects of reducing screen time, present opportunities for implementing health promotion interventions like encouraging balanced media use, foster offline social interactions, and support mental health literacy. Further research is needed to examine the direction and causality of these associations, requiring more longitudinal research, and future interventions examining social media use should consider sex-specific approaches.

## Data Availability

No datasets were generated or analysed during the current study.

## Abbreviations

HUNT: Trøndelag Health Study
QoL: Quality of life
HRQoL: Health related quality of life
ILC: Inventory of Life Quality
HSCL-10: Hopkins Symptoms Checklist-10
SES: Socio-economic status
ANOVA: Analysis of variance
